# Exosomes derived from M2 macrophage promote HUVECs proliferation, migration and tube formation *in vitro*

**DOI:** 10.1038/s41598-025-03113-5

**Published:** 2025-05-22

**Authors:** Yiwen Xue, Jingru Chen, Xin Sun, Chuanchao Su, Siyu Fan, Xiao Song, Runzhi Deng, Jing Hao

**Affiliations:** 1 Nanjing Stomatological Hospital, Affiliated Hospital of Medical School, Institute of Stomatology, Nanjing University, Nanjing, 210008 China; 2 Changsha Medical University, Changsha, 410219 China

**Keywords:** Cancer, Oncology

## Abstract

Angiogenesis is one of the hallmarks of solid tumors. Exosomes are extracellular vesicles, which are involved in multiple stages of tumor progression. The role of macrophage-derived exosomes in angiogenesis in oral squamous cell carcinoma (OSCC), where macrophages are crucial components of the tumor microenvironment (TME), remains to be explored. The M2 macrophages and microvessels in OSCC were stained by immunohistochemistry. M2 macrophages were induced *in vitro* and exosomes were extracted by gradient centrifugation using ultracentrifugation. Fluorescence confocal microscopy was used to observe the uptake of M2 macrophage-derived exosomes by human umbilical vein endothelial cells (HUVECs). CCK-8 proliferation assay, TRANSWELL migration assay and tube formation assay were used to detect the effects of M2 macrophage-derived exosomes on the proliferation, migration and tube formation of HUVECs. Immunohistochemical study showed that M2 macrophage infiltration was positively correlated with microvessel density in OSCC. We induced M2 macrophages *in vitro* and successfully extracted its exosomes. The cellular internalization of fluorescence-labeled exosomes by HUVECs was dynamically monitored via laser scanning confocal microscopy. The M2 macrophage-derived exosomes promoted the proliferation, migration, and tube-forming ability of HUVECs. The results showed that M2 macrophage-derived exosomes could promote the proliferation, migration and tubulation of HUVECs.

**Supplementary Information:**

The online version contains supplementary material available at 10.1038/s41598-025-03113-5.

## Introduction

The incidence of oral cancer accounts for the majority of head and neck malignancies^1^. According to statistics, in 2020, there were 377,000 new patients with oral cancer and about 177,000 new deaths due to oral cancer worldwide^2^. OSCC is a malignant tumor originating from the epithelium, accounting for about 90% of oral cancer cases, and it is characterised by high recurrence and mortality rates, with lymphatic and haematogenous metastasis being its main modes of metastasis^3^.

In 1971, Judah Folkman firstly proposed that angiogenesis plays a supportive role in the growth of tumors, and that the nutrients and oxygen required for tumor growth come from the blood vessels, and angiogenesis is closely related to tumor proliferation, invasion and metastasis. In OSCC, Sasahira et al. found a positive correlation between microvessel density and T stage, clinical stage, local recurrence and distant metastasis of the tumor, suggesting that angiogenesis plays a contributory role in the progression of OSCC^4^.

Tumor cells, endothelial cells, fibroblasts, macrophages, and the extracellular matrix surrounding or infiltrating the tumor form the TME, which is involved in tumor angiogenesis by secreting cytokines and growth factors^5^. Macrophages are one of the major immune cells in the TME and have attracted research interest due to their different roles at different stages of tumor progression. Macrophages in the TME are derived from bone marrow-derived monocytes and, in the early stages of tumor progression, differentiate predominantly into M1 macrophages, which exert anti-tumor effects. As the tumor progresses, influenced by cytokines secreted by malignant cells, macrophages polarise towards the M2 type, which promotes tumor progression by facilitating proliferation, metastasis and angiogenesis^6^. Current research indicates that the infiltration of M2 macrophages is positively correlated with the poor prognosis of various tumors, including OSCC^7^. M2 macrophages exhibit secretory functions for pro-angiogenic cytokines and inflammatory mediators, including VEGF, PIGF and IL-1β. These molecular effectors drive tumor neovascularization through dual mechanisms of endothelial cell proliferation potentiation and apoptosis suppression^8^. Anti-angiogenic therapeutics have emerged as a strategically significant modality in oncology. Contemporary research paradigms extend beyond VEGF pathway inhibition to encompass combinatorial approaches integrating anti-angiogenic agents with immunotherapeutic strategies. However, therapeutic limitations persist, predominantly attributable to intrinsic or adaptive drug resistance mechanisms. Microenvironmental plasticity constitutes a critical resistance determinant, mediated through TME remodeling, cancer stem cell (CSC) populations, and immunosuppressive niche formation. Notably, genomic profiling reveals that angiogenic pathway dysregulation occurs preferentially in tumor stromal compartments rather than malignant epithelial cells, suggesting stromal-targeted interventions may enhance therapeutic efficacy^9,10^. Therefore, more tumor angiogenesis mechanisms need to be explored in order to provide new therapeutic targets or avoid drug resistance to anti-angiogenesis therapy. Although it has been shown that M2 macrophage infiltration in OSCC correlates with the microvessel density of the tumor^11^, however, the specific mechanisms regarding the promotion of angiogenesis by M2 macrophages in OSCC remain unelucidated.

Exosomes are nanoscale extracellular vesicles (with diameters of approximately 30–150 nanometers) derived from endosome structures. They achieve information exchange and material transfer between cells by delivering various bioactive components including functional proteins, genetic material, and lipid molecules to target cells.These vesicular entities exhibit particular significance in oncogenesis and malignant progression, functioning as molecular conduits that coordinate TME remodeling, metastatic niche formation, and therapeutic resistance development^12^. Tumor cell derived exosomes can promote tumor proliferation, invasion and metastasis, and at the same time, tumor cell derived exosomes can act on macrophages in the TME, promoting their polarisation towards M2 macrophages^13^.Previous studies have shown that endothelial cells are able to take up tumor cell-derived exosomes via endocytosis, initiating the tumor angiogenic signalling pathway and promoting tumor angiogenesis, and similar reports have been made in the field of OSCC^14^.

Other cells in the TME also have the ability to generate exosomes, and these nanoscale carriers are involved in the pathological process of tumor development through multiple molecular mechanisms^15^. Previous studies have shown that infiltration of M2 macrophages is positively correlated with the microvessel density of the tumor, and the same is seen in OSCC; however, the role of their secreted exosomes in OSCC angiogenesis has not yet been elucidated. Therefore, the present study focused on investigating the effects of M2 macrophage exosomes on endothelial cells, aiming to lay the foundation for the effects of M2 macrophage exosomes on angiogenesis in OSCC.

## Results

### M2 macrophage infiltration positively correlates with microvessel density in OSCC

**Fig. 1:**
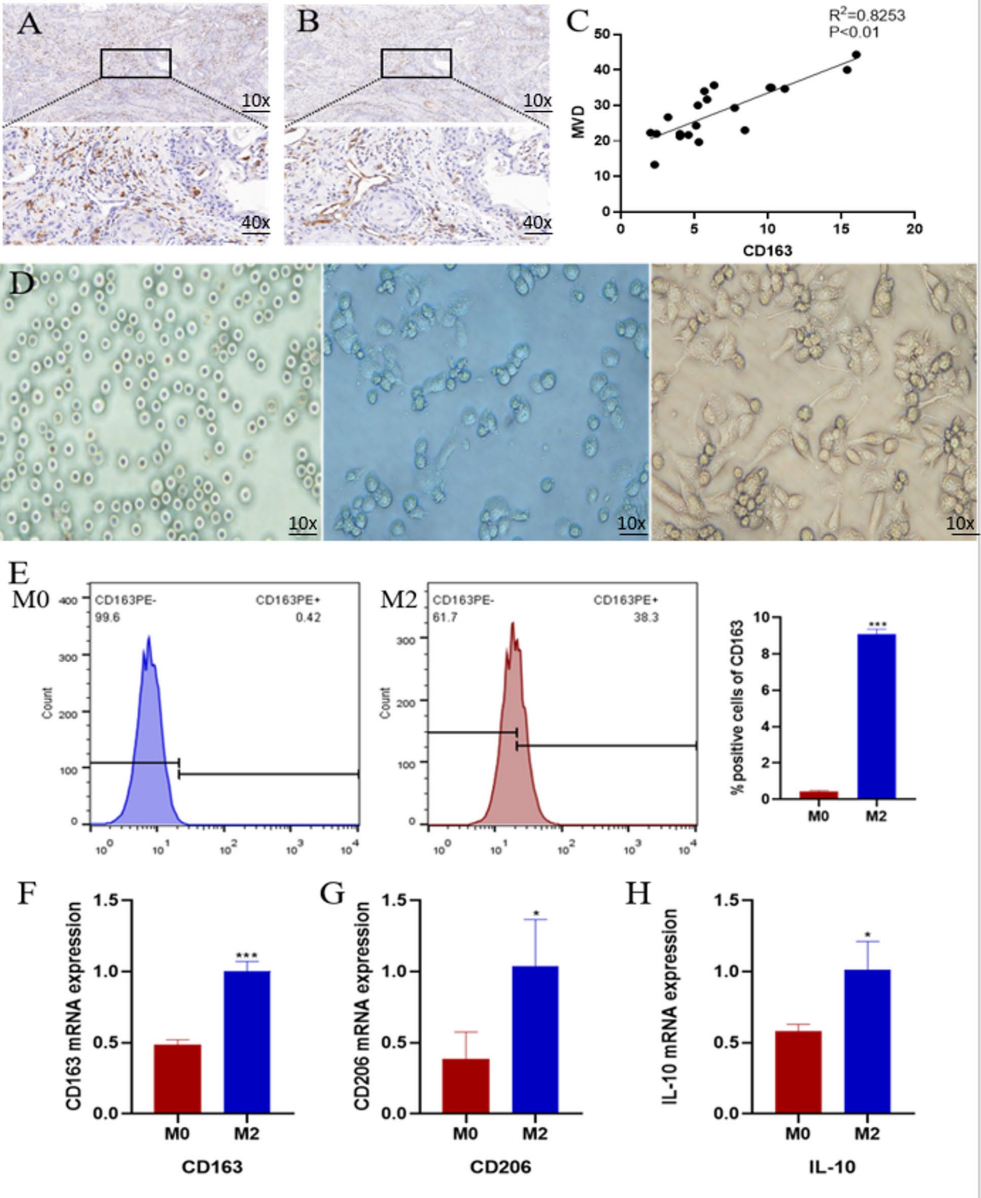
**Relationship between M2 macrophages and OSCC MVD and induction of M2 macrophages* in vitro***. (**A**-**B**) Microscopic observation of the morphology of M2 macrophages and microvessels in OSCC. (**C**) Correlation between M2 macrophage counts and microvessel density in OSCC. (**D**) Morphological changes in THP-1 cells induced by PMA + IL-4. (E) Comparison of cell surface CD163 expression after combined induction with PMA + IL-4 and PMA alone. (F-H) Expression of CD163, CD206, and IL-10 in cells after combined induction of PMA and IL-4. An asterisk (*) indicates *P* < 0.05, two asterisk (**) indicates *P* < 0.01, three asterisks (***) indicate *P* < 0.001, four asterisks (****) indicate *P* < 0.0001, ns indicate *P* > 0.05, ns indicates no significant difference.

Microscopically, CD163-positive and CD31-positive cells are seen to exhibit yellow or brownish-yellow granules(Fig. 1.**A&B**). For microscopic counting of M2 macrophages, three areas of positive expression were randomly selected under low magnification and subsequently counted under high magnification, and the average of the three fields of view was the count of M2 macrophages. The counting of microvessel density was performed in the same way as the counting of M2 macrophages, wherein microvessels were determined according to weidner’s microvessel density counting method(Vascular quantification followed stringent immunohistochemical criteria: CD31-positive endothelial cell aggregates were deemed countable microvessels when morphologically distinct from adjacent tumor parenchyma and stromal components, irrespective of luminal status. Exclusion criteria encompassed (1) vasculature within peritumoral stromal interface zones and sclerotic tumor regions, (2) muscularized vasculature (arterioles/venules), and (3) conduits exhibiting luminal diameters exceeding 8 endothelial cell units). Correlation analysis between M2 macrophage infiltration and microvessel density showed a significant positive correlation**(**Fig. 1.**C)**. This suggests that in OSCC, infiltration of M2 macrophages in the tumor tissue may potentially correlate with tumor angiogenesis.

### Induction of M2 macrophages *in vitro*

Through the combined induction of PMA + IL-4, THP-1 cells changed from rounded suspension to flattened adherent wall with pseudopods protruding in all directions under the microscope**(**Fig. 1.**D)**, consistent with the morphology of M2 macrophages described in the literature^16^. Meanwhile, flow cytometry results showed that the expression of CD163 on the surface of M2 macrophages induced by the combination of PMA + IL-4 was significantly higher compared with that of M0 macrophages induced by PMA alone**(**Fig. 1.**E)**. Meanwhile, qPCR results showed that the expression of genes such as CD163, CD206 and IL-10 in M2 macrophages induced by PMA + IL-4 combination was significantly higher than that in M0 macrophages induced by PMA alone**(**Fig. 1.**F-H)**. Of course, the differences in both flow cytometry and qPCR results were statistically significant. The changes in cell morphology after PMA + IL-4 induction were observed microscopically, which, together with the results of flow cytometry and qPCR, indicated that we successfully induced M2 macrophages *in vitro*.

### Characterization of exosomes

**Fig. 2:**
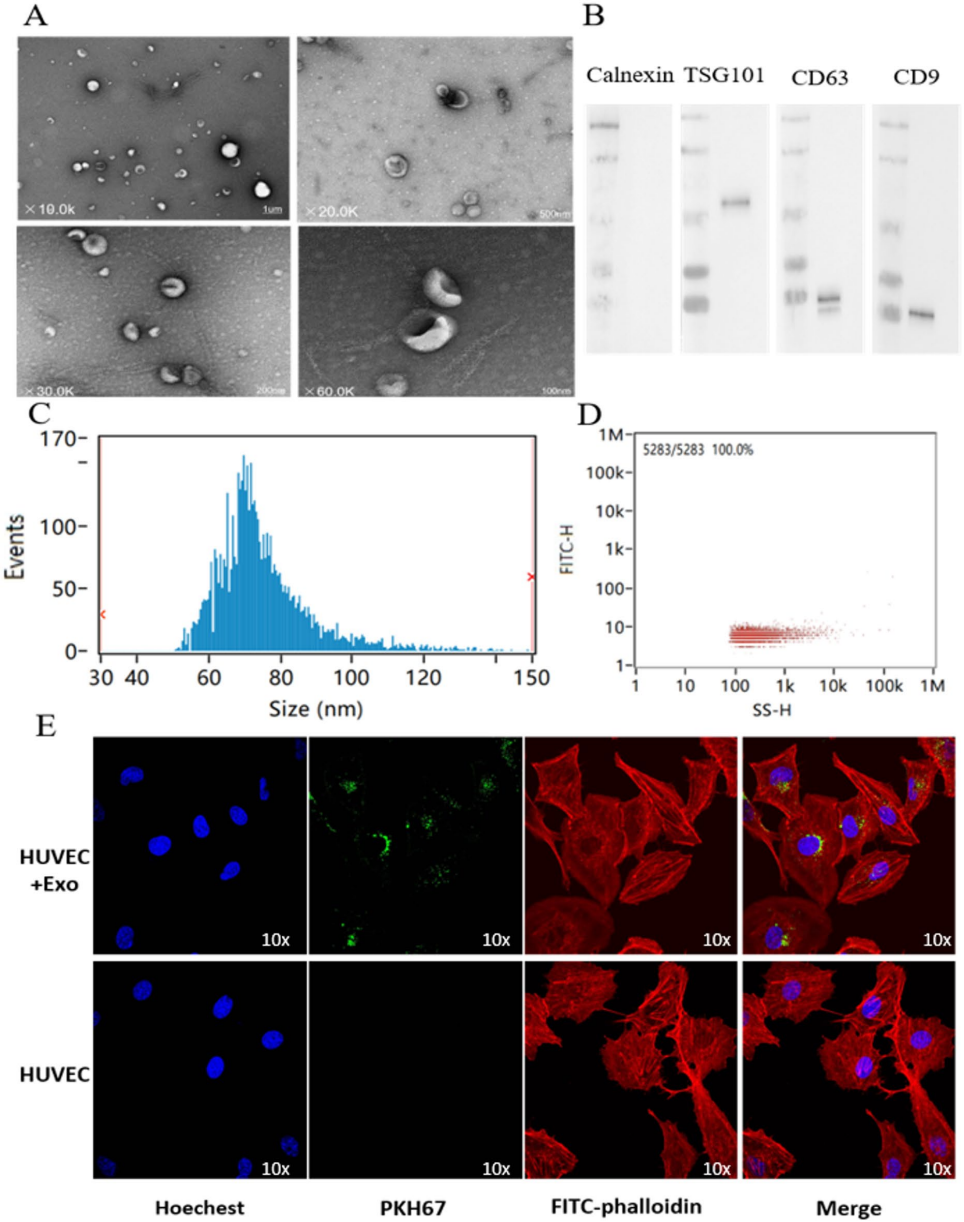
**Identification of M2 macrophage-derived exosomes and their co-culture with HUVEC.** (**A**) Microscopic structure of exosomes (scale bar: 1 mm, 500 nm, 200 nm, 100 nm) in the transmission electron microscope. (**B**) Expression of exosome surface positive marker proteins TSG101, CD63, CD9, and negative marker protein Calnexin. (**C**) Exosome particle size distribution. (**D**) Exosome concentration determination diagram. (**E**) Fluorescence confocal microscopy of PKH67-labelled exosomes co-cultured with HUVEC. HUVEC nuclei were stained with Hoechst (blue), HUVEC Cytoskeleton were stained with FITC-Phalloidin (red), M2 macrophage-derived exosomes were labelled with PKH67 (green).

The exosomes were dissolved in PBS and the exosomal proteins were subsequently extracted, the BCA standard curve was plotted, the concentration of exosomes was determined and the amount of exosomes extracted was calculated.Transmission electron microscopy revealed exosomes as bilayered membranous structures in the form of round or ovoid vesicles**(**Fig. 2.**A)**. NTA analysis showed that the particle size of exosomes was concentrated in the range of 60–100 nm, with an average particle size of 75.09 nm**(**Fig. 2.**C)**. Flow cytometry was used to detect The concentration of exosomes (Fig. 2.**D**), the measured exosome concentration was 7.86E + 11 Particles/mL.Research literature reports that exosomes are extracellular vesicles with a particle size of 30–150 nm, and the particle size of the exosomes we extracted is consistent with literature reports^17^. TSG101, CD63, and CD9 are all common and typical positive markers of exosomes, while Calnexin is a negative marker of exosomes. Western blot analysis confirmed the presence of exosomal markers TSG101, CD63, and CD9 in the isolated vesicles, while calnexin, an endoplasmic reticulum protein serving as a negative control for exosomal purity, was undetectable in the exosome fraction. **(**Fig. 2.**B)**. Guidelines for exosome identification published by the International Society for Extracellular Vesicles state that exosome identification can be accomplished by combining two or more methods. Combined with TEM, NTA and WB results, we were able to confirm the successful extraction of exosomes.

### HUVECs uptake of M2 macrophage exosomes by endocytosis

Microscopically, the exosomes were stained green, the nuclei of HUVECs were stained blue, and the cytoskeleton was stained red. After 24 h of co-incubation of the labelled exosomes with HUVECs, green exosomes were seen scattered around the nuclei of HUVECs under the microscope**(**Fig. 2.**E)**. This suggests that M2 macrophage exosomes are able to be taken up by HUVECs via endocytosis.

### M2 macrophage exosomes promote angiogenesis *in vitro*

**Fig. 3:**
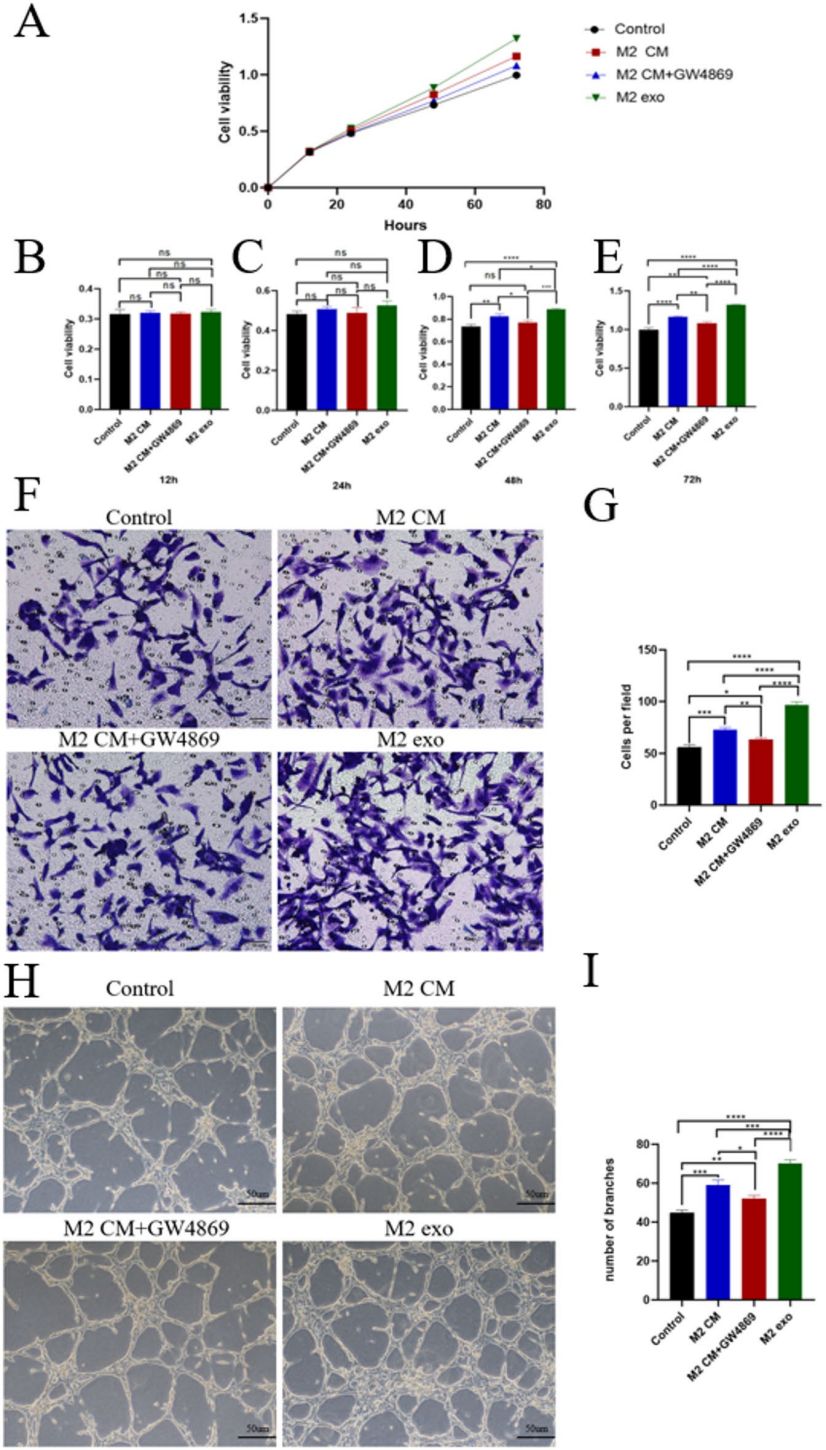
**Effects of M2 macrophage-derived exosomes on HUVEC proliferation**,** migration and tube formation* in vitro***. (**A**-**E**) The CCK-8 method was used to detect changes in the proliferative capacity of HUVEC when four different conditioned media were sub-treated with HUVEC for 12, 24, 48, and 72 h (C, M2 CM, M2 CM + GW4869, M2 Exo). (**F**-**G**) The number of HUVEC migrated after the four conditioned media treatments was observed under the microscope, and the differences in the migration ability of HUVEC after the four groups of conditioned media treatments were compared. (H-I) The number of HUVEC tube-forming tubes after the four conditioned media treatments was counted under the microscope, and the differences in the tube-forming ability of HUVEC after the four groups of conditioned media treatments were compared. An asterisk (*) indicates *P* < 0.05, two asterisk (**) indicates *P* < 0.01, three asterisks (***) indicate *P* < 0.001, four asterisks (****) indicate *P* < 0.0001, ns indicate *P* > 0.05, ns indicates no significant difference.

During tumor progression, angiogenesis requires endothelial cell activation, proliferation and migration. Our experimental results showed that M2 macrophage-derived exosomes demonstrated pro-angiogenic potential *in vitro*. The results of proliferation experiments showed that under four different culture conditions, the proliferation capacity of HUVECs in the M2 exo group was significantly higher than that of the remaining three groups at 48 h**(**Fig. 3.**A-E)**. Similarly, the capacity of HUVECs growth value in the M2 CM group was higher than that in the M2 CM + GW4869 group. The number of HUVEC migrating in different groups was observed under the microscope**(**Fig. 3.**F)**, and the differences in the migration ability of HUVECs in each group were compared. Quantitative analysis of Transwell migration assays revealed that M2 macrophage exosomes induced a statistically superior migratory capacity in HUVECs compared to three control cohorts. Notably, the M2 macrophage exosomes conditioned media (CM) group exhibited enhanced endothelial migration relative to the CM group co-treated with the exosome inhibitor GW4869, suggesting exosome-mediated paracrine signaling as a critical driver of this pro-migratory phenotype**(**Fig. 3.**G)**. This suggests that M2 macrophage exosome significantly enhances HUVECs migration. Tube-forming assay showed that HUVECs co-incubated with M2 macrophage exosomes had significantly enhanced tube-forming ability**(**Fig. 3.**H&I)**. The results of proliferation, migration, and tube formation assays demonstrated that M2 macrophage exosomes promote HUVEC angiogenesis* in vitro*.

## Discussion

Most previous studies on exosomes and tumor angiogenesis have focused on exosomes derived from tumor cells. The innovation point of this study lies in focusing on the role of exosomes derived from M2 macrophages in angiogenesis in the TME. Firstly, the positive correlation between M2 macrophage infiltration and MVD was verified in OSCC clinical samples. Moreover, *in vitro* experiments revealed that M2 macrophage exosomes could be taken up by HUVECs, thereby significantly promoting the proliferation, migration and tube formation ability of HUVECs. It has been clarified that M2 macrophage exosomes are key mediators promoting angiogenesis in the TME, providing new ideas for analyzing the angiogenesis mechanism of OSCC and developing targeted therapeutic strategies. Such as specifically blocking the internalization of M2 macrophage exosomes in endothelial cells of the TME, or taking advantage of the natural targeting property of M2 macrophage exosomes to load chemotherapy drugs to target endothelial cells, etc., thereby specifically inhibiting tumor angiogenesis, in the hope of finding new anti-angiogenic therapies.

With the increasing understanding of the TME, it has been realised that tumors are not just islands of tumor cells alone, but complex microenvironments composed of multiple cells. Macrophages are one of the major infiltrating cells in the TME and are classified into M1 and M2 according to their different states at different stages of the tumor, where M2 macrophages are usually considered as tumor associated macrophages. In solid tumors, infiltration of M2 macrophages is mostly positively associated with poor prognosis due to its ability to promote tumor proliferation, invasion, metastasis and angiogenesis^18^. Similarly, in OSCC, as the malignancy of the tumor increases, the proportion of M2 macrophage infiltration becomes correspondingly higher^19^. In tumors such as breast and ovarian cancers, evidence has confirmed that M2 macrophage infiltration is positively correlated with the microvessel density of tumors, while blocking macrophage recruitment in the TME or polarising macrophages towards M1 can attenuate the angiogenic capacity of tumors, suggesting that M2 macrophages are associated with tumor angiogenesis^20,21^. The present study confirmed a significant positive correlation between M2 macrophages infiltrated in OSCC and the microvessel density of the tumor, which, in combination with the study of Liu et al.^22^, suggests a potential role of M2 macrophages in promoting angiogenesis in oral squamous carcinoma.

In the TME, the polarization of macrophages is a complex process involving multiple mechanisms. Cytokines in the TME, such as IL-4 and IL-13, can promote the polarization of M2 macrophages by activating the STAT6 signaling pathway. At the same time, hypoxic microenvironment of tumor can also promote the polarization of M2 macrophages by up-regulating the expression of VEGF and IL-4R through HIF-1^23^. In this study, IL-4 was used to induce macrophage polarization. After IL-4 induction, the marker (CD163) of M2 macrophages increased significantly.

Tumor angiogenesis is a complex biological process involving a variety of cytokines and signalling pathways. Tumors less than 2 mm in diameter can absorb nutrients from surrounding capillaries to sustain their growth. When a tumor is larger than 2 mm in diameter, neovascularisation is necessary to provide oxygen and nutrient support for growth, so malignant transformation of a tumor is usually accompanied by the onset of a tumor angiogenic switch^24^. Tumor cells initiate tumor angiogenesis by secreting VEGF, which interacts with endothelial cells in the TME and activates the angiogenic signalling pathway^25^. Other cells in the TME participate in the angiogenic process of the tumor by interacting with each other through the release of biologically active substances and activation of signalling pathways. Numerous studies have demonstrated that M2 macrophages are pivotal in tumor angiogenesis. M2 macrophages can secrete VEGF, EGF and other angiogenic factors to directly stimulate the proliferation and migration of endothelial cells. At the same time, M2 macrophages are able to secrete matrix metalloproteinases that degrade the extracellular matrix to reshape the angiogenic microenvironment^26^. Chaudhari et al. showed that M2 macrophages can secrete CCL2, IL-10, IL-13 and VEGF to promote OSCC growth and angiogenesis^27^.

Exosomes mediate intercellular communication by delivering biologically active substances such as genetic material, proteins and lipids from the parental cell to the recipient cell. Exosomes are involved in almost all biological processes, and research on their role in tumor progression has progressed by leaps and bounds in recent years. In terms of tumor angiogenesis, exosomes derived from tumor cells directly activate the proliferation and migration of endothelial cells through the delivery of pro-angiogenic proteins. In addition, miRNAs in tumor exosomes regulate angiogenesis by targeting endothelial cell genes^28^. Exosomes are widely found in blood, urine and various body fluids, but their complex extraction methods and lower yields are the main reasons limiting their application. Despite the development of various exosome extraction methods relying on size-based separation, immunoaffinity capture, and precipitation techniques, however, the gold standard for exosome isolation is still considered to be differential centrifugation^29^. By filtration and differential centrifugation, larger cellular debris, particles and larger extracellular vesicles and apoptotic vesicles can be removed from the cell supernatant, eventually yielding a more purified exosome. By referring to previous studies, M2 macrophage exosomes were finally extracted by differential centrifugation in the present study.Although the extraction of exosomes has been continuously optimised, it is still difficult to completely separate exosomes from other extracellular vesicles and macromolecular protein complexes, so various methods are used to identify the extracted exosomes. Currently, the identification of exosomes mainly relies on morphological features, nanotracer technology to analyse the particle size and immunoprotein blotting technology to analyse the surface marker proteins of exosomes^30^. The guidelines for the identification of exosomes issued by the International Society for Extracellular Vesicles state that only two ways are needed to complete the identification of exosomes, and in this study we used transmission electron microscopy, NTA and WB to prove that we had successfully extracted the exosomes. The cellular internalization of exosomes occurs through heterogeneous endocytic pathways, though the precise molecular choreography governing vesicle-cell interface dynamics remains incompletely resolved. Emerging evidence indicates that exosomal uptake efficiency is principally determined by recipient cell membrane proteome profiles rather than canonical exosome surface markers. Mechanistic studies implicate clathrin-mediated endocytosis and lipid raft-dependent macropinocytosis as predominant routes, with biochemical analyses demonstrating essential roles for tetraspanin superfamily members and integrins in mediating vesicle docking and cytoskeletal remodeling during internalization^31^.Endothelial cells promote tumor angiogenesis by taking up exosomes and activating angiogenic signalling pathways in response to cargoes carried by the exosomes^32^. Previous studies have shown that endothelial cells take up tumor cell-derived exosomes by endocytosis^33^; therefore, in this study, we used PKH67 to label M2 macrophage-derived exosomes and traced them under fluorescence confocal microscopy. After co-culturing the labelled exosomes with endothelial cells, green fluorescent exosomes were seen to appear around the nuclei of HUVECs under fluorescence confocal microscopy, indicating that HUVECs were able to uptake M2 macrophage exosomes by endocytosis.

Endothelial cells are essential in tumor angiogenesis, and proliferation, activation and migration of endothelial cells are necessary steps in angiogenesis. Previous studies have shown that M2 macrophages promote angiogenesis mainly through the interaction of secreted cytokines and growth factors with endothelial cells. M2 macrophages secrete VEGF, PIGF, IL-6, and TNF, and their ability to promote angiogenesis has been demonstrated in other studies. However, compared with the M2 CM + GW4869 group, HUVECs proliferation, migration, and proliferation were significantly enhanced in the M2 CM and M2 exo groups, while the proliferation, migration, and tube-forming capacity of HUVEC in the M2 CM group was significantly lower than that in the M2 exo group. All results showed that M2 macrophage-derived exosomes were able to promote endothelial cell proliferation, migration, and tube-forming capacity* in vitro*, suggesting that they may be potential pro-angiogenic factors.

In the hypoxic state of the TME, exosomes can be abundantly released, mediating dynamic communication between tumor cells and neighboring cells while promoting tumor malignant transformation. Tumor cell-derived exosomes have been shown to play an important role in tumor progression by promoting tumor angiogenesis. The ability of tumor cell-derived exosomes to promote tumor angiogenesis has been demonstrated in tumors such as breast, lung and ovarian cancer, and similar studies have been reported in OSCC^34–37^. Ozel Capik et al. demonstrated that in the OSCC microenvironment, tumor cell-derived exosomes promote angiogenesis via the miR-1825/TSC2/mTOR axis^38^. In addition to tumor cell exosomes, studies have shown that M2 macrophage exosomes can promote tumor proliferation, migration and invasion. Therefore, compared with the study of Ozel Capik et al., we mainly focused on the role of M2 macrophage exosomes in OSCC angiogenesis, in order to further explore the role of M2 macrophage exosomes in tumor progression.

Exosomes promote tumor angiogenesis by releasing a variety of angiogenesis-promoting cargoes to endothelial cells, such as microRNAs, miRNAs and proteins. Exosomes derived from tumor cells facilitate breast cancer angiogenesis by transferring miR-210 to endothelial cells, which suppresses the expression of Ephrin A3 and PTP1B while enhancing VEGF levels and promoting new vascular structure formation^39^. In ovarian cancer, Millimaggi et al. showed that CD147 is overexpressed in tumor cell-derived exosomes and plays an important role in tumor angiogenesis^40^. Exosomes are natural carriers of coding and non-coding RNAs, so they can be used as carriers for tumor-targeting drugs. Exosomes are non-immunogenic and have a natural structure and properties, as well as being nanoscale and carrying a variety of molecules on their surfaces that are able to pass through a variety of biological barriers. Liu et al. demonstrated that the use of exosomal transport of antisense RNA targeting miR-150, which promotes the expression of the angiogenic factor VEGF, significantly reduces VEGF expression and blocks tumor angiogenesis^41^. HGF is known to have pro-angiogenic effects, and Zhang et al. showed that HGF siRNA contained in exosomes could be transferred to gastric cancer cells, thereby inhibiting HGF expression^42^. The above studies indicate that exosomes not only play an important role in tumor progression, but also have great potential for application in tumor therapy. Macrophage-derived exosomes can similarly promote tumor angiogenesis by delivering substances such as nucleic acids to endothelial cells.

In the present study, we demonstrated that M2 macrophage-derived exosomes can promote the proliferation and migration of HUVECs and tube formation *in vitro*, but the specific mechanism still needs to be further investigated, and the above findings point out the direction for our next research. Similarly, this study lacks animal experiments to demonstrate the effect of M2 macrophage exosomes on angiogenesis in OSCC *in vivo*, which also needs to be further investigated.

## Materials and methods

### Immunohistochemistry

This study was approved by the Ethics Committee of Nanjing Stomatological Hospital. Tumor tissues from 20 patients with OSCC were selected from the biological sample bank of Nanjing Stomatological Hospital to be made into paraffin sections. The sections were stained by immunohistochemistry, and CD31(Abcam ab9498) was used to label endothelial cells in the tumor tissues, and CD163(Abcam ab182422) was used to label M2 macrophages in the tumor tissues. Staining for CD163 and CD31 was observed under the microscope.

### Cell lines and cell culture

THP-1 cells were purchased from Shanghai Cell Bank, Chinese Academy of Sciences and cultured with RPMI-1640 (Gibco, USA)containing 10% foetal bovine serum(Gibco, USA). THP-1 cells were first treated with 100ng/ml PMA(Sigma-Aldrich P1585)for 24 h to induce differentiation into M0 macrophages, followed by discarding the PMA and rinsing three times with PBS(Gibco, USA) to wash away excess PMA and cell debris. Then the treatment was continued using 20ng/ml IL-4 (genscript, z02925, China)for 48 h to induce differentiation of M0 macrophages to M2 macrophages.HUVECs were purchased from keyGEN BioTECH and cultured in F12 K(Gibco, USA)medium containing 10% FBS.Both THP-1 and HUVECs were incubated in an incubator containing 5% carbon dioxide.

### qPCR and flow cytometry

To demonstrate our success in inducing M2 macrophages, flow cytometry and qPCR were used to validate the cells after induction. CD163 is one of the typical M2 macrophage marker molecules, in this study, we used PE-CD163(BioLegend, 326505,USA) antibody to stain the induced cells, and subsequently examined the difference in CD163 expression between the cells induced by the combination of PMA + IL-4 and the cells induced by PMA alone.

Assays for gene expression are another way to validate that CD163, CD206 and IL-10 are all marker genes for M2 macrophages. In the present study, RNA was extracted from PMA + IL-4 co-induced and PMA-induced cells using an RNA extraction kit(Vazyme, Nanjing, China), followed by transcription of RNA into cDNA, and finally qPCR was used to detect the expression of the three genes mentioned above in the two groups of cells. Primer sequences for CD163, CD206 and IL-10 are shown in Table 1.

**Table 1:** Sequences of primers used in quantitative real-time PCR (qRT-PCR) analysis.

Genes	Primer sequence	
	Forward	Reverse
CD163	ATCAACCCTGCATCTTTAGACA	CTTGTTGTCACATGTGATCCAG
CD206	GGGTTGCTATCACTCTCTATGC	TTTCTTGTCTGTTGCCGTAGTT
IL-10	GTTGTTAAAGGAGTCCTTGCTG	TTCACAGGGAAGAAATCGATGA
GAPDH	CCAGGTGGTCTCCTCTGA	GCTGTAGCCAAATCGTTGT

### Exosome extraction and purification

When M2 macrophage differentiation was complete, the original supernatant was discarded and the dish was subsequently rinsed three times with PBS. Then DMEM medium containing exosom-free serum was added to the petri dish and the supernatant was collected after continuing the incubation for 48 h.

Exosomes were extracted using differential centrifugation. Firstly, the cell supernatant was centrifuged at 3000 rpm for 30 min to remove larger cellular debris from the supernatant; next, the above centrifuged supernatant was filtered using a 0.22 μm filter to remove larger particles from the supernatant; subsequently, the filtered supernatant was centrifuged at 17,000 rpm for 30 min for the removal of other extracellular vesicles and other complexes from the supernatant. Finally, the supernatant obtained from the above high-speed centrifugation was centrifuged at 110,000 rpm for 80 min, and the supernatant was subsequently discarded to obtain the exosomes at the bottom of the centrifuge tube. The extracted exosomes were dissolved in 200ul of PBS and mixed well. A 50ul suspension of exosomes was taken, to which an equal amount of protein lysate(Vazyme, Nanjing, China) was added to extract the proteins of the exosomes, and subsequently the concentration of the exosomes was detected using the BCA kit(Vazyme, Nanjing, China), and the BCA standard curve was plotted to calculate the amount of the extracted exosomes.

### Exosome identification

The identification of exosomes relies on morphological features, nanoparticle tracking technology to analyse particle size and protein immunoblotting technology to analyse marker proteins on the exosome surface. The morphology of exosomes was observed under transmission electron microscope(Hitachi, Chiyoda, Japan) at 100 Kv and the morphological features were photographed and recorded. Dynamic light scattering with a particle size analyser (NanoFCM, Xiamen, China) was employed to determine the size distribution of exosomes. Western blotting was conducted to detect the expression of exosome surface-enriched proteins CD9, CD63, and TSG101, using calnexin as a negative marker protein.

### Exosome internalisation assay

Fluorescence confocal experiments were used to observe the interaction between M2 macrophage-derived exosomes and HUVECs. The exosome suspension was labelled with fluorescent labelled probe PKH67(Merck, Darmstadt, Germany), PBS was rinsed to remove excess dye, and the HUVECs suspension was inoculated into six-well plates, and the labelled exosomes were added for co-culturing after the cells were attached to the wall. Following 24-hour co-culture, the uptake of exosomes by HUVECs was visualized using a fluorescence confocal microscope (FV3000, Olympus, Tokyo, Japan).

### Proliferation, migration and tube formation experiments

The experiment was divided into 4 groups, namely control group(C), M2 macrophage conditioned medium group(M2 CM), M2 macrophage conditioned medium + GW4869 group(M2 CM + GW4869) and M2 macrophage exosome group(M2 exo). The control group was blank medium.M2 CM was obtained by adding medium containing 10% exosome-free serum to M2 macrophages, collecting the supernatant after 48 h and centrifuging it at high speed. Medium containing 10% exosome-free serum was added to M2 macrophages, followed immediately by the addition of GW4869 (MCE, Princeton, New Jersey, USA) to the medium, after which the supernatant was collected and the conditioned medium for the M2 CM + GW4869 group could be obtained by high-speed centrifugation. The M2 exo group is the extracted M2 macrophage exosome.

In this study, the CCK-8 method was used to detect the proliferative capacity of HUVECs. After HUVECs was treated with the four sets of conditioned media for 12, 24, 48, and 72 h, 10 µl of CCK-8 solution(Vazyme, Nanjing, China) was added to each well and continued to be incubated in the incubator for 1–4 h. Subsequently, the OD at 450 nm was measured using an enzyme marker.

The TRANSWELL migration assay was used to detect the difference in migration ability of HUVECs treated with different conditioned media. HUVECs were treated for 48 h as per the above grouping, and TRANSWELL chambers were placed in 24-well plates with 100 µl of treated HUVECs suspension in the upper chamber and 500 µl of medium containing 10% FBS in the lower chamber, followed by continued incubation for 24 h in an incubator. At the end of the culture, the cells in the lower chamber were fixed with paraformaldehyde and stained with crystal violet, followed by counting the number of HUVECs migrating to the lower chamber under the microscope, and three randomly selected fields of view were averaged.

Finally, tube-forming assay was used to detect the difference in tube-forming ability of HUVECs in each group. 50 µl of matrix gel(Corning, USA) was added to each well of a 96-well plate and placed in an incubator to allow it to solidify. Next, HUVECs suspensions treated with the above four sets of conditioned media were prepared, and about 10,000 cells were added to each well, which were then placed in the incubator for further incubation. 6 h later, the tube formation of each set of HUVEC was observed under the microscope again, and the tubules were counted under the microscope, with three randomly selected fields of view counted in each well, and the average value was taken. All cell experiments *in vitro* were repeated at least three times to ensure the stability of the experimental results.

### Statistical analysis

Statistical analyses were conducted using GraphPad Prism 8 (GraphPad Software, Inc., La Jolla, CA, USA). Data were presented as mean ± standard deviation. Pearson correlation analysis was used to assess correlations between two groups, and independent samples t-tests were performed for pairwise comparisons. All datasets underwent normality testing; when data exhibited normal distribution and homogeneous variances, differences among the four groups were evaluated using one-way analysis of variance (ANOVA).

## Electronic supplementary material

Below is the link to the electronic supplementary material.

Supplementary Material 1

Supplementary Material 2

Supplementary Material 3

Supplementary Material 4

## Data Availability

All data generated or analyzed during this study are included in this article. Further inquiries can be directed to the corresponding author.

## References

[1] Johnson, D. E. et al. Head and neck squamous cell carcinoma. *Nat. Rev. Dis. Primers*. **6**, 92. 10.1038/s41572-020-00224-3 (2020). 33243986 10.1038/s41572-020-00224-3 PMC7944998

[2] Sung, H. et al. Global Cancer statistics 2020: GLOBOCAN estimates of incidence and mortality worldwide for 36 cancers in 185 countries. *CA Cancer J. Clin.* **71**, 209–249. 10.3322/caac.21660 (2021). 33538338 10.3322/caac.21660

[3] Fan, T. et al. NUPR1 promotes the proliferation and metastasis of OSCC cells by activating TFE3-dependent autophagy. *Signal. Transduct. Target. Ther.* **7**, 130. 10.1038/s41392-022-00939-7 (2022). 35462576 10.1038/s41392-022-00939-7 PMC9035452

[4] Sasahira, T. et al. MIA-dependent angiogenesis and lymphangiogenesis are closely associated with progression, nodal metastasis and poor prognosis in tongue squamous cell carcinoma. *Eur. J. Cancer*. **46**, 2285–2294. 10.1016/j.ejca.2010.04.027 (2010). 20570137 10.1016/j.ejca.2010.04.027

[5] Xiao, Y. & Yu, D. TME as a therapeutic target in cancer. *Pharmacol. Ther.* **221**, 107753. 10.1016/j.pharmthera.2020.107753 (2021). 33259885 10.1016/j.pharmthera.2020.107753 PMC8084948

[6] Boutilier, A. J. & Elsawa, S. F. Macrophage polarization States in the TME. *Int. J. Mol. Sci.* **22**10.3390/ijms22136995 (2021). 10.3390/ijms22136995 PMC8268869 34209703

[7] Dan, H. et al. RACK1 promotes cancer progression by increasing the M2/M1 macrophage ratio via the NF-kappaB pathway in OSCC. *Mol. Oncol.* **14**, 795–807. 10.1002/1878-0261.12644 (2020). 31997535 10.1002/1878-0261.12644 PMC7138402

[8] Lin, E. Y. et al. Macrophages regulate the angiogenic switch in a mouse model of breast cancer. *Cancer Res.* **66**, 11238–11246. 10.1158/0008-5472.CAN-06-1278 (2006). 17114237 10.1158/0008-5472.CAN-06-1278

[9] Cascone, T. et al. Upregulated stromal EGFR and vascular remodeling in mouse xenograft models of angiogenesis inhibitor-resistant human lung adenocarcinoma. *J. Clin. Invest.* **121**, 1313–1328 (2011). 21436589 10.1172/JCI42405 PMC3070607

[10] Huijbers, E. J. M., Beijnum, J. R. V., Thijssen, V. L., Sabrkhany, S. & Griffioen, A. W. Role of the tumor stroma in resistance to anti-angiogenic therapy. *Drug Resist. Updates*. **25**, 26–37 (2016). 10.1016/j.drup.2016.02.002 27155374

[11] Sun, H. et al. TGF-beta1/TbetaRII/Smad3 signaling pathway promotes VEGF expression in OSCC tumor-associated macrophages. *Biochem. Biophys. Res. Commun.* **497**, 583–590. 10.1016/j.bbrc.2018.02.104 (2018). 29462614 10.1016/j.bbrc.2018.02.104

[12] Zhang, L. & Yu, D. Exosomes in cancer development, metastasis, and immunity. *Biochim. Biophys. Acta Rev. Cancer*. **1871**, 455–468. 10.1016/j.bbcan.2019.04.004 (2019). 31047959 10.1016/j.bbcan.2019.04.004 PMC6542596

[13] Yin, Y. et al. Colorectal Cancer-Derived small extracellular vesicles promote tumor immune evasion by upregulating PD-L1 expression in tumor-Associated macrophages. *Adv. Sci. (Weinh)*. **9**, 2102620. 10.1002/advs.202102620 (2022). 35356153 10.1002/advs.202102620 PMC8948581

[14] Ludwig, N. et al. Tumor-derived exosomes promote angiogenesis via adenosine A(2B) receptor signaling. *Angiogenesis* **23**, 599–610. 10.1007/s10456-020-09728-8 (2020). 32419057 10.1007/s10456-020-09728-8 PMC7529853

[15] Wu, J. et al. M2 Macrophage-Derived exosomes facilitate HCC metastasis by transferring alpha(M) beta(2) integrin to tumor cells. *Hepatology* **73**, 1365–1380. 10.1002/hep.31432 (2021). 32594528 10.1002/hep.31432 PMC8360085

[16] Orecchioni, M., Ghosheh, Y., Pramod, A. B. & Ley, K. Macrophage polarization: different gene signatures in M1(LPS+) vs. Classically and M2(LPS–) vs. Alternatively activated macrophages. *Front. Immunol.* **10**10.3389/fimmu.2019.01084 (2019). 10.3389/fimmu.2019.01084 PMC6543837 31178859

[17] Doyle, L. & Wang, M. Overview of extracellular vesicles, their origin, composition, purpose, and methods for exosome isolation and analysis. *Cells* **8**10.3390/cells8070727 (2019). 10.3390/cells8070727 PMC6678302 31311206

[18] Pan, Y., Yu, Y., Wang, X. & Zhang, T. Tumor-Associated macrophages in tumor immunity. *Front. Immunol.* **11**, 583084. 10.3389/fimmu.2020.583084 (2020). 33365025 10.3389/fimmu.2020.583084 PMC7751482

[19] Chaudhari, N. et al. Evaluation of density of tumor-associated macrophages using CD163 in histological grades of OSCC, an immunohistochemical study. *J. Oral Maxillofacial Pathology: JOMFP* **24**, 577 . 10.4103/jomfp.JOMFP_109_20 PMC8083437 33967504

[20] Stockmann, C. et al. Deletion of vascular endothelial growth factor in myeloid cells accelerates tumorigenesis. *Nature* **456** (2008). 10.1038/nature07445 PMC3103772 18997773

[21] Yeo, E. J. et al. Myeloid WNT7b mediates the angiogenic switch and metastasis in breast Cancer. *Cancer Res.* **74**, 2962–2973 (2014). 24638982 10.1158/0008-5472.CAN-13-2421 PMC4137408

[22] Liu, D., Sun, W., Zhang, D. & Yin, J. Long noncoding RNA GSEC promotes neutrophil inflammatory activation by supporting PFKFB3-involved glycolytic metabolism in sepsis. (2021). 10.1038/s41419-021-04428-7 PMC8671582 34907156

[23] Chaurasia, A. et al. Tumour-Associated macrophages in OSCC. *Oral Dis.*10.1111/odi.15265 (2025). 39846431 10.1111/odi.15265

[24] Plate, K. H., Scholz, A. & Dumont, D. J. Tumor angiogenesis and anti-angiogenic therapy in malignant gliomas revisited. *Acta Neuropathol.* **124**, 763–775. 10.1007/s00401-012-1066-5 (2012). 23143192 10.1007/s00401-012-1066-5 PMC3508273

[25] Claesson-Welsh, L. & Welsh, M. VEGFA and tumour angiogenesis. *J. Intern. Med.* **273**, 114–127. 10.1111/joim.12019 (2013). 23216836 10.1111/joim.12019

[26] Mantovani, A., Marchesi, F., Malesci, A., Laghi, L. & Allavena, P. Tumour-associated macrophages as treatment targets in oncology. *Nat. Reviews Clin. Oncol.* **14**, 399–416. 10.1038/nrclinonc.2016.217 (2017). 10.1038/nrclinonc.2016.217 PMC5480600 28117416

[27] Chaudhari, N. et al. Evaluation of density of tumor-associated macrophages using CD163 in histological grades of OSCC, an immunohistochemical study. *J. Oral Maxillofacial Pathol.* **24**10.4103/jomfp.JOMFP_109_20 (2020). 10.4103/jomfp.JOMFP_109_20 PMC8083437 33967504

[28] Ye, Z. W., Yu, Z. L., Chen, G. & Jia, J. Extracellular vesicles in tumor angiogenesis and resistance to anti-angiogenic therapy. *Cancer Sci.* **114**, 2739–2749. 10.1111/cas.15801 (2023). 37010195 10.1111/cas.15801 PMC10323098

[29] Greening, D. W., Xu, R., Ji, H., Tauro, B. J. & Simpson, R. J. A protocol for exosome isolation and characterization: evaluation of ultracentrifugation, density-gradient separation, and immunoaffinity capture methods. *Methods Mol. Biol.* **1295**, 179–209. 10.1007/978-1-4939-2550-6_15 (2015). 25820723 10.1007/978-1-4939-2550-6_15

[30] Zhu, L. et al. Isolation and characterization of exosomes for cancer research. *J. Hematol. Oncol.* **13**, 152. 10.1186/s13045-020-00987-y (2020). 33168028 10.1186/s13045-020-00987-y PMC7652679

[31] Tkach, M. & Théry, C. Communication by extracellular vesicles: where we are and where we need to go. *Cell* **164**, 1226–1232 (2016). 26967288 10.1016/j.cell.2016.01.043

[32] Zhuang, G., Wu, X., Jiang, Z., Kasman, I. & Ferrara, N. Tumour-secreted miR-9 promotes endothelial cell migration and angiogenesis by activating the JAK-STAT pathway. *EMBO J.* **31**, 3513–3523 (2013). 10.1038/emboj.2012.183 PMC3433782 22773185

[33] Nazarenko, I. et al. Cell surface tetraspanin Tspan8 contributes to molecular pathways of exosome-induced endothelial cell activation. *Cancer Res.* **70**, 1668–1678. 10.1158/0008-5472.CAN-09-2470 (2010). 20124479 10.1158/0008-5472.CAN-09-2470

[34] Jung, K. O., Youn, H., Lee, C. H., Kang, K. W. & Chung, J. K. Visualization of exosome-mediated miR-210 transfer from hypoxic tumor cells. *Oncotarget* **8** (2016). 10.18632/oncotarget.14247 PMC5354779 28038441

[35] Yi, H., Ye, J., Yang, X. M., Zhang, L. W. & Chen, Y. P. High-grade ovarian cancer secreting effective exosomes in tumor angiogenesis. *Int. J. Clin. Experimental Pathol.* **8**, 5062 (2015). PMC4503072 26191200

[36] Hsu, Y. L. et al. Hypoxic lung cancer-secreted exosomal miR-23a increased angiogenesis and vascular permeability by targeting prolyl hydroxylase and tight junction protein ZO-1. Oncogene (2017). 10.1038/onc.2017.105 28436951

[37] Ludwig, N. et al. TGFbeta(+) small extracellular vesicles from head and neck squamous cell carcinoma cells reprogram macrophages towards a pro-angiogenic phenotype. *J. Extracell. Vesicles*. **11**, e12294. 10.1002/jev2.12294 (2022). 36537293 10.1002/jev2.12294 PMC9764108

[38] Capik, O., Gumus, R. & Karatas, O. F. Hypoxia-induced tumor exosomes promote angiogenesis through miR‐1825/TSC2/mTOR axis in OSCC. *Head Neck*. **45**, 2259–2273. 10.1002/hed.27460 (2023). 37449548 10.1002/hed.27460

[39] Jia, Y., Chen, Y., Wang, Q., Jayasinghe, U. & Zhou, J. Exosome: emerging biomarker in breast cancer. *Oncotarget* **8**, 41717–41733 (2017). 28402944 10.18632/oncotarget.16684 PMC5522217

[40] Millimaggi, D. et al. Tumor vesicle-associated CD147 modulates the angiogenic capability of endothelial cells. *Neoplasia* **9**, 349–357. 10.1593/neo.07133 (2007). 17460779 10.1593/neo.07133 PMC1854851

[41] Liu, Y., Zhao, L., Li, D., Yin, Y. & Zhang, Y. Microvesicle-delivery miR-150 promotes tumorigenesis by up-regulating VEGF, and the neutralization of miR-150 attenuate tumor development. *Protein Cell.* **4**, 932–941 (2013). 24203759 10.1007/s13238-013-3092-z PMC4875402

[42] Zhang, H. et al. Exosomes serve as nanoparticles to suppress tumor growth and angiogenesis in gastric cancer by delivering hepatocyte growth factor SiRNA. *Cancer Sci.* (2018). 10.1111/cas.13488 PMC5834801 29285843

