# Supplementary figures and images for: Exosomes derived from M2 macrophage promote HUVECs proliferation, migration and tube formation in vitro
Source: Sci Rep. 2025 May 22;15:17876. doi: 10.1038/s41598-025-03113-5 (PMC12098727; doi:10.1038/s41598-025-03113-5)

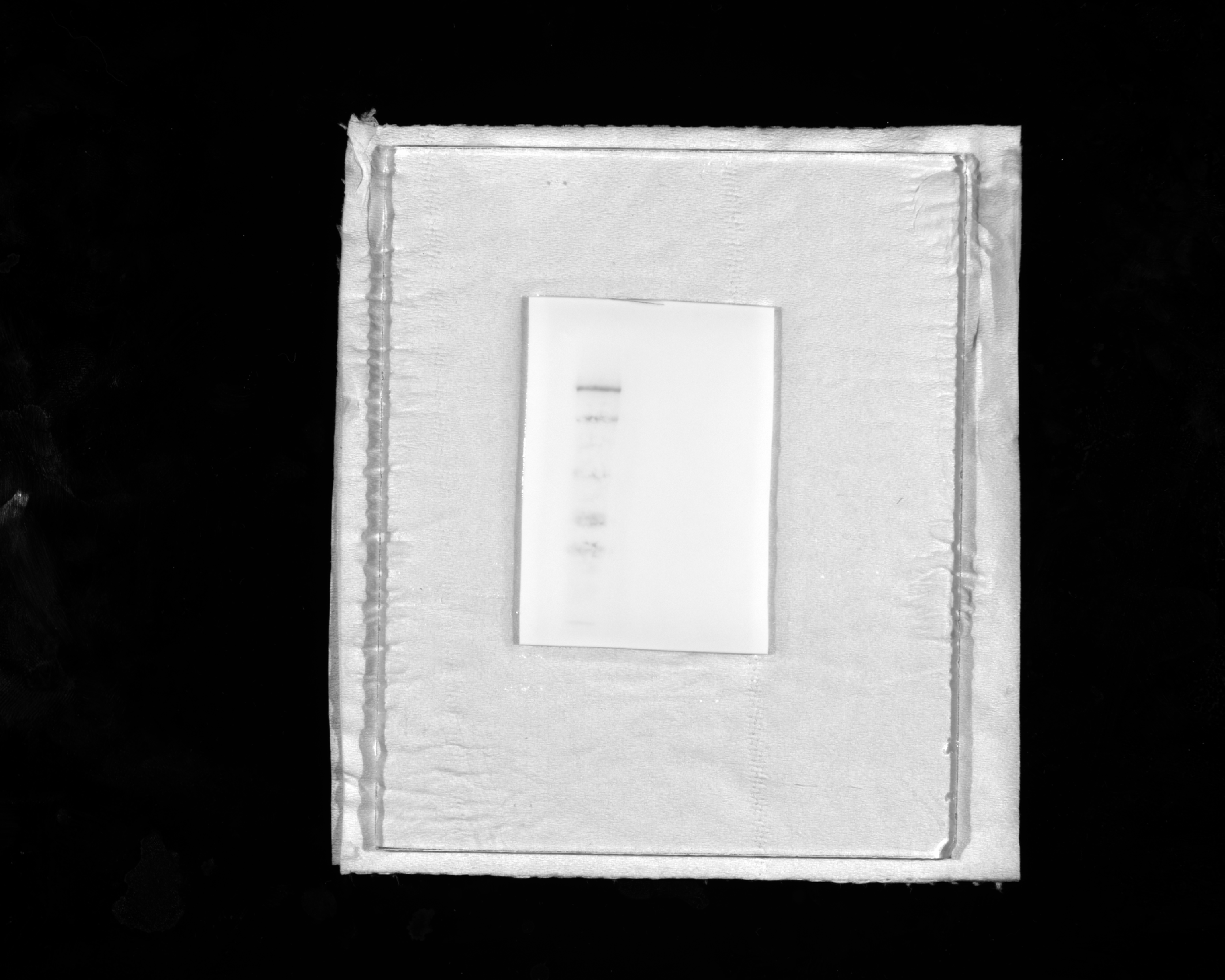

Supplement: Supplementary file 1 — Supplementary Material 1 [file 41598_2025_3113_MOESM1_ESM.tif]

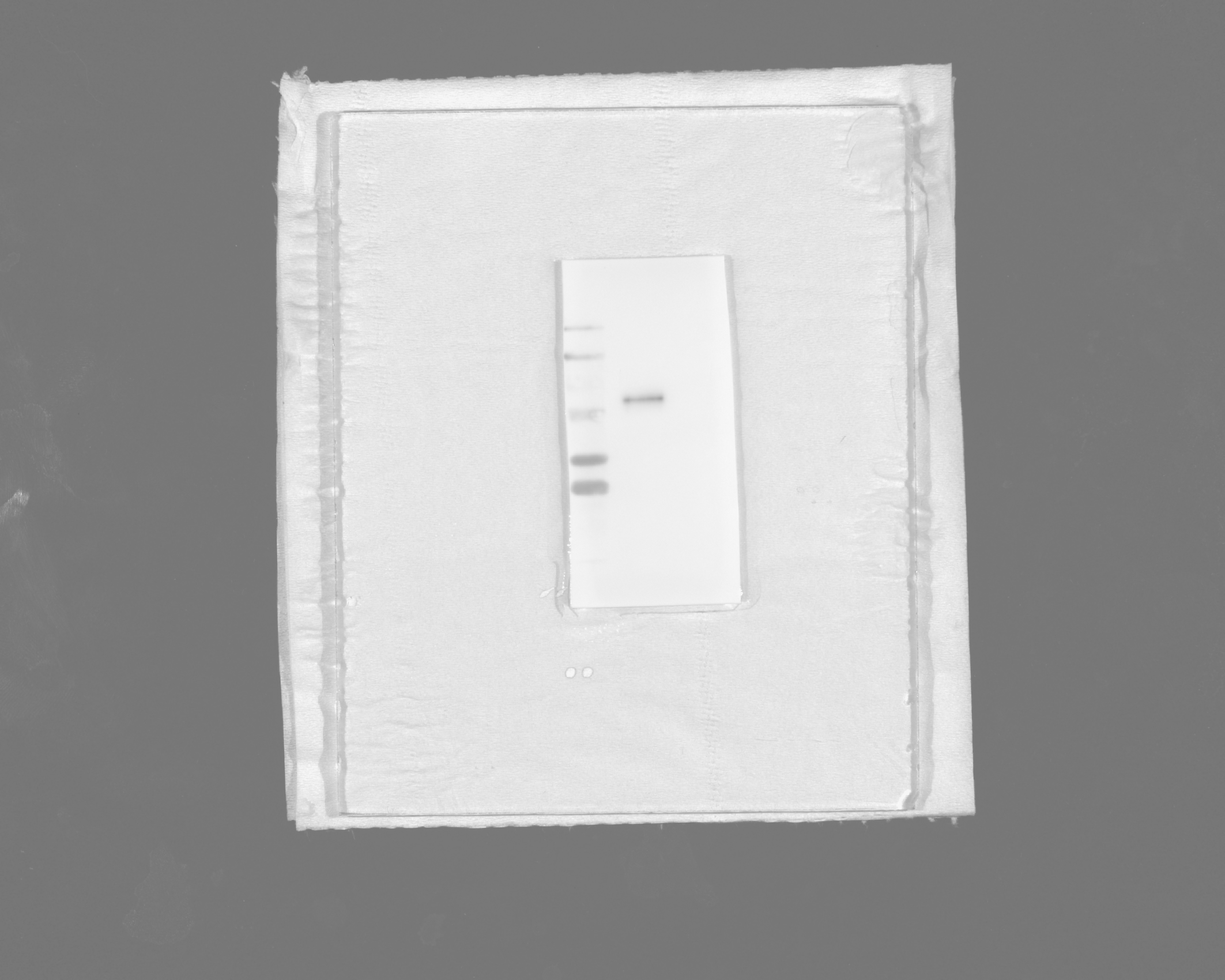

Supplement: Supplementary file 2 — Supplementary Material 2 [file 41598_2025_3113_MOESM2_ESM.tif]

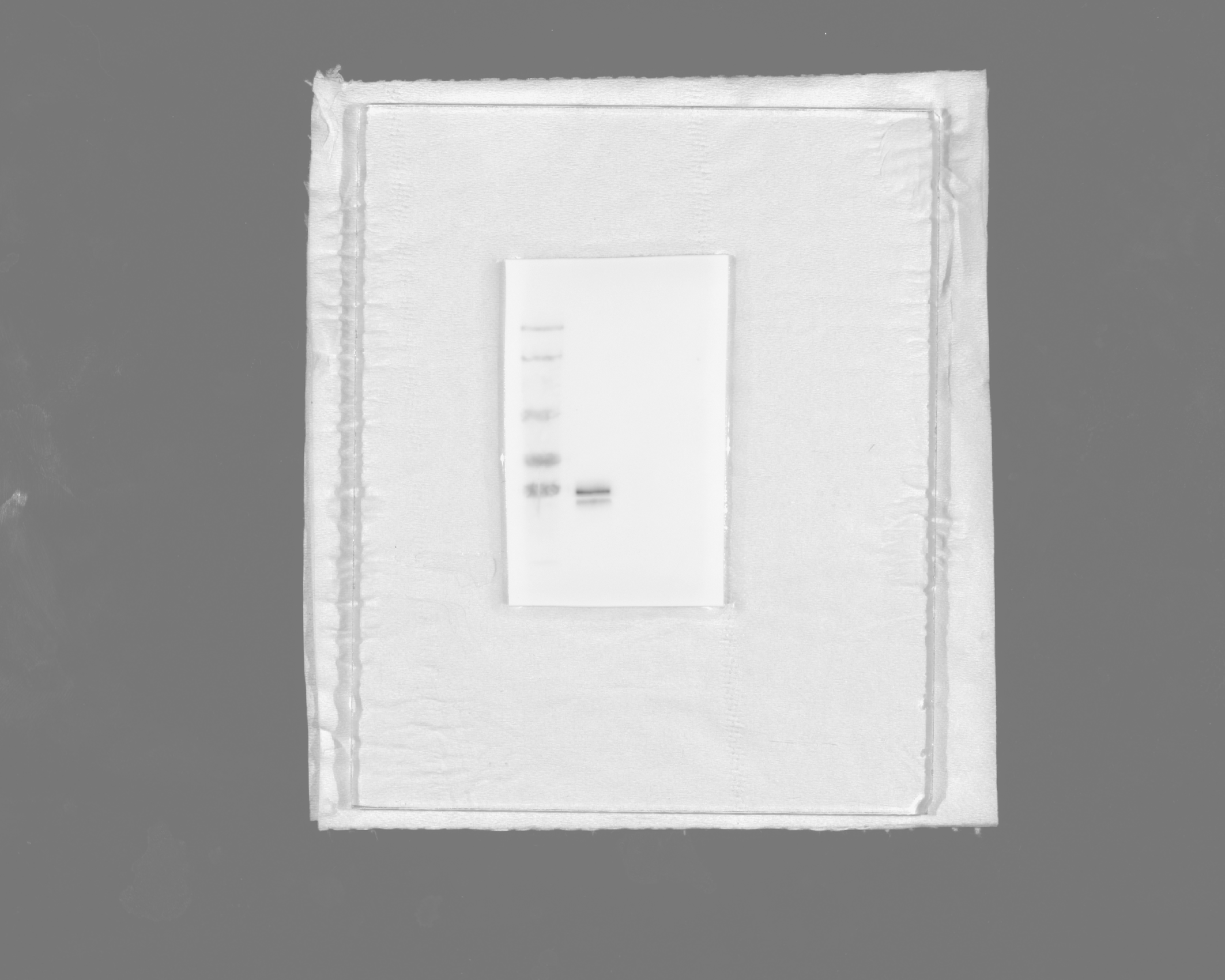

Supplement: Supplementary file 3 — Supplementary Material 3 [file 41598_2025_3113_MOESM3_ESM.tif]

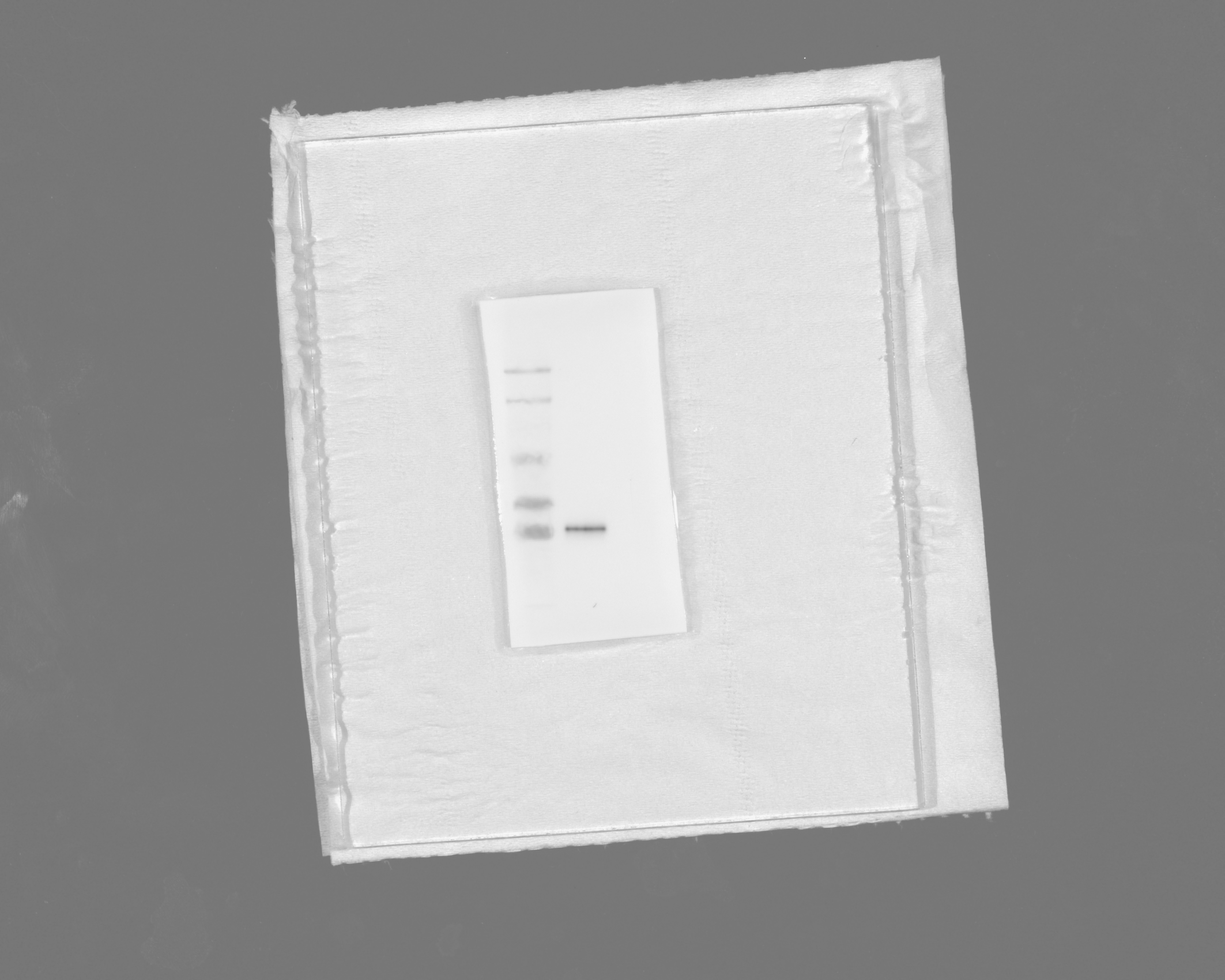

Supplement: Supplementary file 4 — Supplementary Material 4 [file 41598_2025_3113_MOESM4_ESM.tif]
